# Performance Analysis of the IEEE 802.11ax MAC Protocol for Heterogeneous Wi-Fi Networks in Non-Saturated Conditions

**DOI:** 10.3390/s19071540

**Published:** 2019-03-29

**Authors:** Kyu-haeng Lee

**Affiliations:** Department of IoT, Soonchunhyang University, Asan-si 336-745, Chungcheongnam-do, Korea; leekh@sch.ac.kr

**Keywords:** 802.11ax, multi-user, non-saturation

## Abstract

The IEEE 802.11ax standard, which realizes multi-user transmission based on orthogonal frequency division multiple access (OFDMA), has been highlighted as a key technology to meet high future demand for Wi-Fi systems. Since this standard is still being developed, performance analysis through mathematical modeling is of paramount importance; however, existing studies have several limitations. Firstly, most of these consider only the saturation network throughput, whereas for 802.11ax in particular, the access delay of the nodes needs to be studied carefully, since they no longer acquire the medium independently but depend solely on the access point’s (AP’s) schedule. Secondly, since the network performance may be affected to a greater extent by legacy nodes than by 802.11ax nodes, it is desirable to consider various heterogeneous cases of networks with existing legacy nodes. In this paper, a new analytical framework for the 802.11ax MAC protocol is provided. Markov-chain-based models are developed to represent the behavior of the 802.11ax nodes, and both non-saturated traffic conditions and co-existence with the legacy nodes are considered. Through both analysis and MATLAB simulations, it is shown that the proposed model accurately evaluates the throughput and the delay performance under various network conditions.

## 1. Introduction

Wi-Fi has emerged as a key off-loading technology to alleviate the data explosion in cellular networks, since it provides many advantages such as ease of installation, free Internet access, and high data rates. The number of devices and users of Wi-Fi is continuously growing; research results from industry show that there will be around 500 million Wi-Fi hotspots by 2021 and that the amount of mobile traffic will also increase significantly [1]. Dense deployment scenarios will become more prevalent than they are at present, and users are likely to expect a high level of Wi-Fi service even in these environments.

It has been observed that multi-user (MU) transmission, which can simultaneously transmit different data streams to multiple users, has great potential to meet the challenge of demand for future Wi-Fi systems. IEEE 802.11ac [2], the first Wi-Fi standard supporting MU transmission based on multi-input multi-output (MIMO) technology, has been developed and deployed, but this has several disadvantages that greatly limit the gain in MU transmission, such as a high channel state information (CSI) acquisition overhead [3] and lack of MU transmission in the uplink (UL) direction. In particular, the efficient processing of UL traffic has recently become an important user requirement (e.g., private streaming services have rapidly become popular around the world), and therefore to keep pace with this new trend in Internet access, it is essential to provide appropriate UL MU transmission in Wi-Fi networks.

A subsequently introduced standard called 802.11ax [4] realizes UL MU transmission based on orthogonal frequency division multiple access (OFDMA), which divides a transmission into several sub-channels and simultaneously transmits data streams for multiple users. One cumbersome issue in employing OFDMA in Wi-Fi involves achieving fine transmission synchronization between transmitting stations (STAs) that have been configured to operate in a fully distributed manner [5]. To overcome this, 802.11ax allows access points (APs) to control all OFDMA transmissions in both downlink (DL) and UL directions. The AP collects the transmission intention from STAs, and schedules UL transmissions for the STAs by allocating OFDMA resources to them. More importantly, it triggers the actual UL transmission by the STAs by broadcasting a ‘*trigger frame*’ (TF) to the network. However, this results in 802.11ax STAs having access to the channel in a completely different way from existing legacy nodes, and in unavoidable changes to the MAC layer.

Since the standard is still being developed, performance analysis through mathematical modeling is of paramount importance, and researchers have published numerous analytical studies on the 802.11ax MAC protocol [6,7,8,9,10]. Unfortunately, most of these provide only limited analytical results, making it difficult to obtain a complete understanding of 802.11ax. They primarily consider only the saturation network throughput, and focus on determining the theoretical capacity limit; however, the network is not always saturated, meaning that this is not practical. In particular, the access delay of 802.11ax STAs should be studied carefully, since these do not acquire the medium independently, but depend solely on the AP’s schedule. To properly analyze both the delay and the throughput, a more realistic traffic model should be used.

Secondly, most of these studies do not consider the issue of co-existence with non-802.11ax STAs. Although 802.11ax will soon be launched on the market, it is impossible to expect that all wireless devices will be equipped with 802.11ax functionality; instead, it is very likely that 802.11ax and legacy devices will co-exist for a very long time. In this situation, the network performance may be affected more strongly by the legacy devices than by the 802.11ax devices, and in particular it may be limited by severe unfairness among heterogeneous nodes. Hence, for a more accurate system analysis, it is desirable to consider various heterogeneous networks with existing legacy devices.

This paper provides a new analytical framework for the 802.11ax MAC protocol. Analytical models based on Markov chains are developed to capture the behavior of 802.11ax STAs, and to take into account the interaction between the AP and the legacy STAs. All these models consider non-saturated traffic conditions in practice by employing different data frame arrival rates for each model; in particular, the rate of TF arrival is used in these models to investigate the impact of the UL OFDMA schedule of the AP on the performance. To provide a complete understanding of 802.11ax OFDMA, both the DL and UL OFDMA transmissions are taken into account in the design of the model. Using both analysis and MATLAB simulations, it is shown that the proposed model accurately evaluates the throughput and the delay performance in various network conditions where legacy STAs and 802.11ax STAs co-exist.

The remainder of this paper is organized as follows. Section 2 summarizes research results related to this paper, and the MU transmission scheme used in 802.11ax is presented in Section 3. The proposed analytical framework is described in Section 4, and Section 5 presents the results of a performance evaluation. Finally, the paper is concluded in Section 6.

## 2. Related Work

Even before the development of 802.11ax, several mechanisms had been proposed for the adoption of OFDMA in Wi-Fi networks [11,12,13,14]. Kwon et al. proposed to exploit OFDMA in the process of channel access by conducting backoff in both the time and frequency domains [11]. The proposed protocol reduces the frame collision rate, and thus improves channel use for the legacy systems. Fallah et al. designed a two-step transmission process for the MAC layer, one that allocates OFDMA resources and another for actual data transmission, to allow the use of OFDMA in Wi-Fi networks [12]. Unlike the aforementioned works, in which the main features are conducted by STAs, the following studies resemble the current 802.11ax protocol, since the AP acts as a coordinator for OFDMA transmission [13,14]. Several control frames were designed by Lou et al. to allow the AP to efficiently control both UL and DL OFDMA transmissions [13], and Deng et al. directly exploited the legacy point coordination function (PCF) for the same purpose [14]. Most of these works, however, do not comply with the OFDMA MU of 802.11ax, and thus they are not sufficient to fully analyze the performance of 802.11ax.

Several recent studies have not only provided advanced features for improving the performance of 802.11ax, but have also presented numerical analysis based on mathematical modeling [6,7,8,9,10]. Lanante et al. provide an analytical model for 802.11ax UL OFDMA with saturated network throughput [6]. Naik et al. consider diverse OFDMA scenarios by applying the latest 802.11ax amendment in their model [7]. The MAC protocol designed by Lee et al. extends the use of the contention window for OFDMA resource allocation [8]. Bellata et al. determine the optimal number of active STAs that maximizes the system throughput, under the assumption that both MU-MIMO and OFDMA MU are available [9]. Khorov et al. investigate an effective contention window size to balance the transmission opportunities of 802.11ax and legacy stations [10]. Unfortunately, most of these studies consider only the saturated system throughput, and the analysis of the access delay for UL OFDMA is not taken into account. Furthermore, all nodes of networks are assumed to be capable of 802.11ax, which limits the use of the analysis results.

As wireless communication technologies have become more diverse, various heterogeneous network scenarios have received much interest from researchers and been considered in many studies (e.g., directional/omni-directional antennas [15], different antenna array configurations [16,17], 802.11ax/non-802.11ax [10]). One common issue in these scenarios is that severe unfairness may limit the network performance (in particular that of wireless nodes with high potentials, such as OFDMA-capable nodes and many-antenna-equipped nodes.) To solve this, Takai et al. propose a new carrier sensing mechanism to exploit the gain of directional antennas in mobile ad hoc networks where nodes equipped with directional antennas and omni-directional antennas co-exist [15]. Lin et al. develop an interference alignment and cancellation-based MAC protocol to maximize the number of concurrent transmissions in heterogeneous MIMO networks where nodes equipped with different antenna array configurations can co-exist [16]. Along this line, Babich et al. claim to use an antenna array adaptively to enhance gains of multiple packet transmissions. Meanwhile, several scheduling algorithms have been developed for both increasing the OFDMA gain and handling the unfairness problem [10,18,19]. An adaptive Enhanced Distributed Channel Access (EDCA) mechanism for OFDMA resource scheduling is proposed by Karthik et al. [18], and Deng et al. and Khorov et al. exploit a multi-level priority scheme for the 802.11ax random access by using different EDCA parameter sets [10,19]. In this paper, the proposed models will be used to describe a possible unfairness problem that may happen in heterogeneous networks where the 802.11ax and legacy nodes co-exist, and through a set of simulations, it will be shown that applying EDCA appropriately can help to alleviate the fairness issue.

## 3. OFDMA Multi-User Transmissions in 802.11ax

In this section, the basic mechanism of the OFDMA MU transmission of 802.11ax is described, as the background to this study. Please note that since the exact details of the operation of the 802.11ax MAC layer are still under discussion, this paper focuses on the main design principle.

OFDMA works by dividing a transmission across several sub-channels, which are referred to as resource units (RUs). According to the current version of 802.11ax, 20 MHz, 40 MHz, 80 MHz and 160 MHz Wi-Fi channels can be divided into 9, 18, 37, and 74 RUs, respectively, and each 802.11ax STA is assigned to a subset of RUs to transmit and receive frames [4].

As mentioned earlier, the biggest change to the 802.11ax MAC protocol compared to the previous generations of the 802.11 family is the use of a TF [4,20]. In essence, the role of a TF is to give meta-information for the subsequent actual OFDMA data transmission. It conveys several items of data, such as which STAs to communicate with, which RUs the STAs should use, and the duration of the corresponding OFDMA transmission. Most importantly, it helps to realize the synchronization of transmission among distributed STAs.

**Figure 1:**
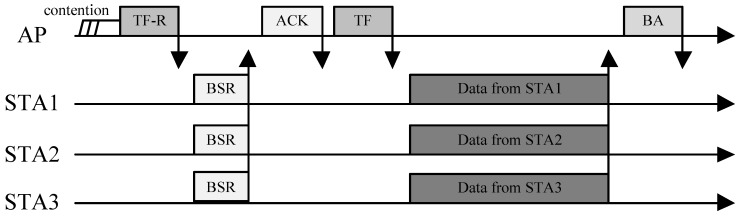
**Example of an UL OFDMA transmission in 802.11ax.** The AP first collects the data transmission intention from STAs via buffer state requests (BSR), and then schedules an UL OFDMA transmission. The actual UL transmission of STAs is initiated with the subsequent TF sent by the AP.

Figure 1 shows an example of the operation of an UL OFDMA transmission in 802.11ax; the DL OFDMA case is omitted, since it is so straightforward. From the figure, we can see that the protocol consists of two steps. In the first step, the AP collects the data transmission intention from the STAs and allocates RUs to them via its own scheduling policy. It is worth noting that this process is performed in a random-access manner. It begins when the AP broadcasts a TF-R (a TF for random access, a variant of a TF [21]), and upon receiving the TF-R, STAs ask the AP to allocate RUs if they have data to send. To do this, 802.11ax introduces another new frame called a buffer state request (BSR).

**Figure 2:**
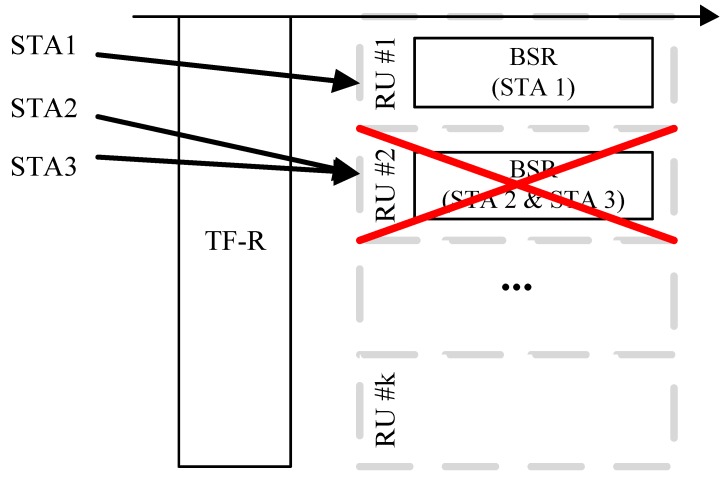
**Contention for OFDMA RU.** STAs contend for RUs in a random-access manner. In this example, only STA 1 succeeds in RU allocation.

When sending a BSR, each STA selects one RU and transmits the BSR to the AP using the selected RU. This is very similar to the random channel access of the distributed coordination function (DCF) in conventional Wi-Fi MAC protocols. The main difference here is that STAs compete for RUs in the frequency domain. If the STA succeeds in sending a BSR without collision (i.e., no other STAs have picked up the same RU), it is assigned a subset of the RUs, which are used for actual data transmission from the AP. Figure 2 shows an example. In this example, STA 2 and STA 3 fail to be allocated RUs, since they collide in the BSR transmission, and will not have the opportunity for transmission until they succeed in sending a BSR. After RU allocation has been carried out, the actual UL OFDMA transmission begins with a TF sent by the AP. Finally, the AP sends a block ACK (BA) to the STAs, reporting the transmission result. Since it may seem somewhat inefficient to make a transmission schedule for each UL transmission, the current amendment allows the resource allocation to run simultaneously with the actual UL data transmission. To do this, only a part of the entire set of RUs is used for BSR transmission, and the rest are used for data transmissions of STAs that have been previously scheduled.

Please note that a higher OFDMA transmission gain can be achieved by allocating RUs to STAs or STA groups appropriately for satisfying certain objective functions [22,23], such as sum-rate maximization and proportional fair, and it can be further improved by jointly considering MU-MIMO and OFDMA. In this paper, for simplicity equal OFDMA RU allocation is assumed since how to optimally assign STAs to OFDMA RUs is beyond the scope of this work.

## 4. Modeling of the 802.11ax MAC Protocol

### 4.1. Network Model and Assumptions

This paper considers a heterogeneous Wi-Fi network in which one AP, $N_{le}$ legacy STAs (denoted as ‘LE’) and $N_{ax}$ 802.11ax STAs (denoted as ‘AX’) co-exist. *N* also denotes the total number of STAs, i.e., $N=N_{le}+N_{ax}$. Only the AP and the AX STAs are assumed to be equipped with OFDMA functionality, and *K* RUs are available for each OFDMA transmission. It is assumed that the AX STAs request RUs from the AP for each transmission.

All the nodes operate in unsaturated traffic conditions: a frame arrives at the transmission queue of each node in a Poisson manner, with rate $\lambda^{data}$. For the AP, in addition to the data frame, a TF reaches the transmission queue with rate $\lambda^{tf}$, which means that it needs to handle two types of frame: if the frame is a TF, the UL OFDMA process is invoked, and otherwise, depending on the type of the receiving node, a decision is made on whether to use DL OFDMA or not (if it is for a LE STA, it is sent without using OFDMA, and vice versa otherwise.) For simplicity of analysis, it is assumed that a single DL OFDMA frame includes several sub-frames for different AX STAs before it reaches the transmission queue of the AP. More specifically, at least 1 to $B=min(N_{ax},K)$ different sub-frames can be aggregated into a single DL OFDMA frame, and the probability of each case is same as $\frac{1}{B}$.

The same data rate and payload size (denoted as *L*) are assumed, but in the case of the OFDMA frame, the total transmission time increases in proportion to the number of sub-frames within it. For example, when three sub-frames are aggregated into one OFDMA transmission, the transmission time roughly increases three-fold compared to a single non-OFDMA transmission.

The subscripts ‘ap’, ‘le’ and ‘ax’ are used to represent the AP, the LE STA, and the AX STA, respectively, and the superscripts ‘ul-le’, ‘ul-ax’, ‘dl-le’, and ‘dl-ax’ are used for the UL transmission of LE STAs, UL transmission of AX STAs, DL transmission to LE STAs, and DL transmission to AX STAs, respectively. *m* represents the node type, i.e., the AP, the LE STA or the AX STA.

### 4.2. Analytical Model Based on Markov Chains

The channel access process of each node is modeled using a Markov chain. For the AP and the LE STAs, we can use several conventional analytical models [24,25,26,27] that were designed for existing random channel access in DCF. For the AX STAs, however, we cannot directly apply those models, since they access the channel in a completely different way than the AP and the LE STAs. Recall that they have the opportunity for transmission only when the AP broadcasts a TF. The next subsection briefly reviews the analytical model for the AP and LE STAs, and then elaborates the model for the AX STAs in more detail.

#### 4.2.1. Model of the AP and LE STAs

**Figure 3:**
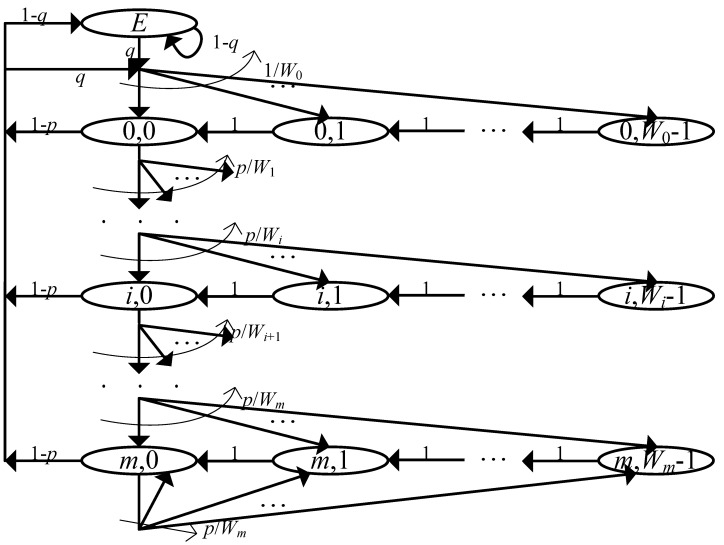
**Model of the AP** **and LE STAs.**

Figure 3 illustrates the model for the AP and LE STAs. In this model, the chain consists of two state groups: the empty-queue state, denoted by *E*, and the backoff states, denoted by $\left(i,w_{i}\right)$ where i∈[0,m] and $w_{i}\in \left[0,W_{i}\right]$. At each backoff stage *i*, a node attempts to transmit when it reaches the (i,0) state, and the access probability τ can be obtained by adding all the stationary probabilities that it is in the (i,0) states. If the transmission is successfully completed, the node goes to the *E* state or $\left(0,w_{0}\right)$ state depending on its queue status; if the queue is empty, then it goes to the *E* state with probability (1−p)(1−q), where *p* represents the probability that the transmitted frame is in collision, and exits from it with probability *q* when a new frame arrives in the queue; otherwise, it goes to $\left(0,w_{0}\right)$ state with probability $\frac{(1-p)q}{W_{0}}$. If the transmitted frame is in collision, the node goes to the $\left(i+1,w_{i+1}\right)$ state with probability $\frac{p}{W_{i+1}}$.

*p* can be easily obtained as follows:

(1) $$\begin{array}{cc} p_{ap} & =1-{\left(1-\tau_{le}\right)}^{N_{le}} \end{array}$$

(2) $$\begin{array}{cc} p_{le} & =1-\left(1-\tau_{ap}\right){\left(1-\tau_{le}\right)}^{N_{le}-1}. \end{array}$$

To represent the non-saturated condition, a parameter *q* is introduced, which denotes the probability that the transmission queue of a tagged node is non-empty. Since a frame arrives at the queue in a Poisson manner, *q* can be estimated [25,26,27,28]:

(3) $$\begin{array}{cc} q_{ap} & =1-e^{-\left(\lambda_{ap}^{data}+\lambda^{tf}\right)E\left[T\right]} \end{array}$$

(4) $$\begin{array}{cc} q_{le} & =1-e^{-\lambda_{le}^{data}E\left[T\right]} \end{array}$$

where E[T] denotes the average slot time, which will be discussed later. Please note that since *q* is related with two cases (i.e., transition from the *E* state and from the (i,0) states), we may need to apply different time values; however in this paper the average value of the whole slot is used, for simplicity of analysis. From Equation (3), we can see that the conjunction of two Poisson arrivals, $\lambda_{ap}^{data}$ and $\lambda^{tf}$, is used for the case of the AP.

Then, from a mathematical computation based on a Markov chain, the access probability τ for both the AP and the LE STAs can be obtained as follows (subscripts are omitted):

(5) $$\begin{array}{c} \tau =\frac{1}{\frac{\left(1-p)(1-q\right)}{q}+\frac{1}{2}+\frac{W_{0}}{2}\left(\frac{1-{\left(2p\right)}^{m}}{\left(1-p)(1-2p\right)}+{\left(2p\right)}^{m}\right)}. \end{array}$$

#### 4.2.2. Model for AX STAs

**Figure 4:**
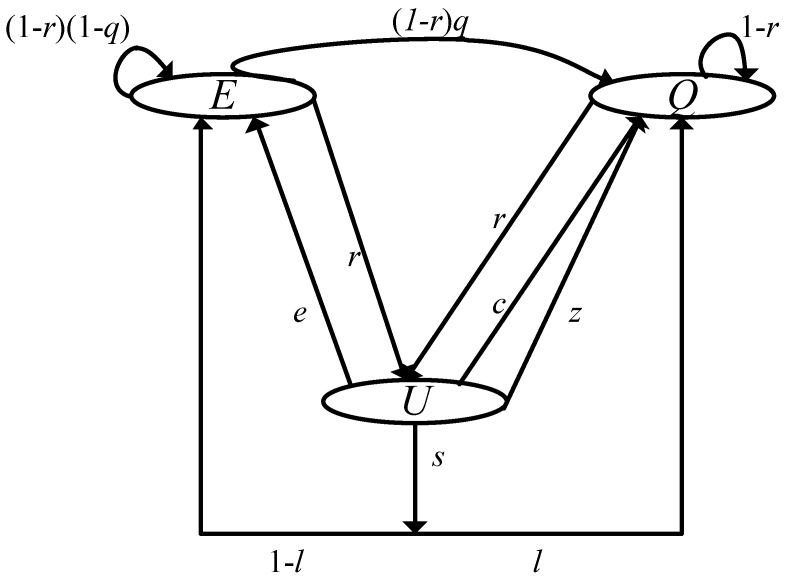
**Model of the AX STAs.** The chain model consists of three states; when receiving a TF from the AP, the AX STAs go into the *U* state.

The new chain model for describing the behavior of AX STAs is illustrated in Figure 4. The chain consists of three states, *E*, *Q*, and *U*. The *E* state is the same as that of the aforementioned model in Figure 3. *Q* represents the state in which a tagged AX STA has a frame to transmit but has not yet received a TF from the AP. *U* models the state in which an UL OFDMA transmission, including RU contention, is initiated by the AP. The notation $b_{E}$, $b_{Q}$ and $b_{U}$ is used to represent the stationary distributions of each state.

We now examine each transition between the states. Although *q* plays the same role as in the previous model, it is associated here only with the *E* and *Q* states. Thus, we have $q_{ax}=1-e^{-\lambda_{ax}^{data}E\left[T^{-ul-ax}\right]}$, where $E[T^{-ul-ax}]$ is the average slot time, except for UL OFDMA transmissions. *r* is enabled only when the AP successfully gains a transmission opportunity and broadcasts a TF. We therefore have:

(6) $$\begin{array}{cc} r & =\tau_{ap}{\left(1-\tau_{le}\right)}^{N_{le}}\cdot \frac{\lambda^{tf}}{\lambda_{ap}^{data}+\lambda^{tf}}. \end{array}$$

After the UL OFDMA transmission is finished, a tagged AX STA returns to either the *E* state or the *Q* state from the *U* state, depending on the transmission result. Four possible transitions can occur: *s*, *c*, *z*, and *e*.

Transition *s* represents the case where a tagged AX STA successfully transmits its frame via UL OFDMA. To achieve this state, two conditions must be met: (i) a frame must exist in the transmission queue at the time of entering the *U* state ($\frac{b_{Q}}{b_{E}+b_{Q}}$), and (ii) at the same time, the tagged STA must win the RU contention. In particular, for the second condition, an additional conditional probability $P_{i}^{\left(n,K\right)}$ is defined to denote that a total of *i* AX STAs win the RU contention, given the condition of *n* contending AX STAs and *K* available sub-channels, and thus these are scheduled for UL OFDMA transmission. Then, *s* can be obtained by adding $P_{i}^{\left(n,K\right)}$ values for all possible combinations of *n* and *i* as follows:

(7) s=bQbEax+bQ∑n=0Nax−1Nax−1nbQbEax+bQnbEaxbEax+bQNax−1−n·∑i=1BPi(n+1,K)1−nCin+1Ci,n≥i∑i=1BPi(n+1,K),otherwise.

The first summation part corresponds to considering all possible combinations of the queue status of $N_{ax}-1$ STAs, while the second part is for adding the probability values. Please note that when n≥i, since some cases that the tagged STA fails in the RU contention are taken into account in computing $P_{i}^{\left(n+1,K\right)}$, those cases should be excluded (i.e., $\left(1-\frac{{}_{n}C_{i}}{{}_{n+1}C_{i}}\right)$). If the STA fails in transmission, it goes into the *Q* state with probability $c=\left(\frac{b_{Q}}{b_{E_{ax}}+b_{Q}}\right)-s$.

One effective way to compute $P_{i}^{\left(n,K\right)}$ is to use dynamic programming; that is, the problem involving the entire set of *K* sub-channels can be solved by dividing it into several smaller overlapping problems. To obtain the value of $P_{i}^{\left(n,K\right)}$, the set of sub-channels is divided into two parts, the first channel and the remaining K−1 channels, and the probability of each is computed. This is repeated for the remaining K−1 channels until *K* is equal to 1. For each iteration, the following two cases should be considered: (i) the case where there is a successful BSR transmission in the first channel, and i−1 successful BSR transmissions occur in the remaining K−1 channels; and (ii) the case where *i* successful BSR transmissions occur only in the remaining K−1 channels. Then, the following recurrence relation is established:

(8) Pi(n,K)=∑j=0nnj1Kj1−1Kn−jP1(j,1)Pi−1(n−j,K−1)+P0(j,1)Pi(n−j,K−1).

**Figure 5:**
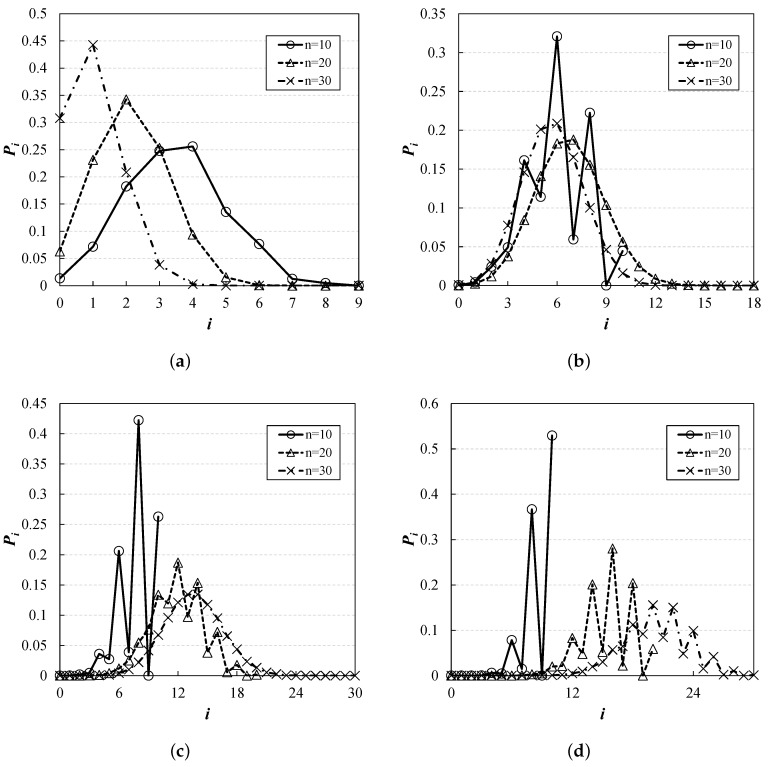
**$P_{i}^{\left(n,K\right)}$ for different values of *n* and *K*, obtained from dynamic programming.** When K>n, the graph looks irregular, but this pattern is correct. (**a**) K=9; (**b**) K=18; (**c**) K=37; (**d**) K=74.

By repeatedly applying Equation (8), we can achieve the desired value. As an example, Figure 5 depicts $P_{i}^{\left(n,K\right)}$ for different values of *n* and *K*. From the results, we observe different probability distributions under various conditions. As expected, if *K* is small compared to *n*, the RU contention is heavier, and as *n* increases, the graph is biased to the left. We also can see that when K>n, the value of certain point *i* is lower or greater than those of the adjacent points, depending on the combination of *n*, *K*, and *i*. As mentioned before, for obtaining $P_{i}^{\left(n,K\right)}$, the following two conditions must be met: (i) certain *i* channels should be associated with just one STA each, and (ii) at the same time, each of the remaining K−i channels must be empty or selected by more than one STAs (i.e., collision). If the number of available channels is large enough (i.e., K>n), it is easy to meet the first condition, on the other hand, it is difficult to meet the second condition in some cases. In other words, even if the number of cases satisfying the first condition is large, the actual probability value could fall significantly due to the second condition. For example, we can see that when K>n, $P_{n-1}^{\left(n,K\right)}$ becomes 0, which is smaller than the surrounding values, causing the curve to appear irregular.

After *s*, depending on its queue status, the tagged AX STA goes into either the *E* state or the *Q* state, with probabilities (1−l) and *l*, respectively. Since *l* is related to only UL OFDMA, we have: $l=1-e^{-\lambda_{ax}^{data}E\left[T^{ul-ax}\right]}$, where $E[T^{ul-ax}]$ is the average UL OFDMA transmission time.

Unlike the previous transitions, i.e., *c* and *s*, a tagged STA may have reached the *U* state from the empty-queue state (i.e., *E*). Please note that in this case, it cannot participate in UL OFDMA transmission, and thus remains silent during the transmission of other STAs. However, a new frame may arrive in the queue during this time, in which case the transition to *Q* through *z* occurs. Using *l*, *z* is expressed as follows:

(9) $$\begin{array}{cc} z & =\frac{b_{E_{ax}}}{b_{E_{ax}}+b_{Q}}\cdot l. \end{array}$$

In contrast, if the queue remains empty, the transition $e=\left(\frac{b_{E_{ax}}}{b_{E_{ax}}+b_{Q}}\right)-z$ is invoked.

Imposing the normalized condition, i.e., $b_{E}+b_{Q}+b_{U}=1$, and with some mathematical manipulation, we have the following stationary distributions of states:

(10) $$\begin{array}{cc} b_{E} & =\frac{r}{r+1}\cdot \frac{s\left(1-l\right)+e}{1-\left(1-r\right)\left(1-q_{ax}^{data}\right)} \end{array}$$

(11) $$\begin{array}{cc} b_{U} & =\frac{r}{r+1}. \end{array}$$

In the next section, the throughput and delay are expressed using the proposed model.

### 4.3. Throughput

The average system throughput is expressed as the sum of successfully transmitted frames of all nodes during the average slot time: (i.e., E[T]): $\frac{E[L_{m}]}{E[T]}$, where $E[L_{m}]$ denotes the average number of successfully transmitted frames of node *m*.

#### 4.3.1. Calculation of E[T]

E[T] is computed by averaging the time of each slot:

(12) $$\begin{array}{cc} E[T] & \\ & =P^{idle}T^{idle}+P^{ul-le}T^{ul-le}+P^{dl-le}T^{dl-le}+P^{col}T^{col}+P^{dl-ax}T^{dl-ax}+P^{ul-ax}T^{ul-ax}\\ & =E\left[T^{-ul-ax}\right]+P^{ul-ax}T^{ul-ax}\\ & =E\left[T^{-ul-ax}\right]+E\left[T^{ul-ax}\right]. \end{array}$$

In the idle slot, neither the AP nor the LE STAs attempt to transmit:

(13) $$\begin{array}{cc} & P^{idle}=\left(1-\tau_{ap}\right){\left(1-\tau_{le}\right)}^{N_{le}}. \end{array}$$

Please note that the AX STAs remain idle until they receive a TF, meaning that Equation (13) does not include any expressions involving the AX STAs.

For the probabilities of UL and DL, we have:

(14) $$\begin{array}{cc} P^{ul-le} & =\left(1-\tau_{ap}\right)N_{le}\tau_{le}{\left(1-\tau_{le}\right)}^{N_{le}-1} \end{array}$$

(15) $$\begin{array}{cc} P^{ul-ax} & =P^{dl}\cdot \frac{\lambda_{tf}}{\lambda_{data}+\lambda_{tf}} \end{array}$$

(16) $$\begin{array}{cc} P^{dl-le} & =P^{dl}\cdot \frac{\lambda_{data}}{\lambda_{data}+\lambda_{tf}}\cdot \frac{N_{le}}{N_{le}+N_{ax}} \end{array}$$

(17) $$\begin{array}{cc} P^{dl-ax} & =P^{dl}\cdot \frac{\lambda_{data}}{\lambda_{data}+\lambda_{tf}}\cdot \frac{N_{ax}}{N_{le}+N_{ax}}. \end{array}$$

where $P^{dl}=\tau_{ap}{\left(1-\tau_{le}\right)}^{N_{le}}$. Please note that except for $P^{ul-le}$, the probability that the AP obtains a transmission opportunity should be considered.

$P^{col}$ is obtained from $P^{col}=1-P^{idle}-P^{dl}-P^{ul-le}$.

We now consider the time parameters. It should be noted when using time parameters that only parameters related to OFDMA are affected by the number of AX STAs actually involved in the transmission; thus, we need to use an average value across all possible cases. Let $T_{i}^{dl-ax}$ and $T_{i}^{ul-ax}$ be the DL and UL OFDMA transmission times for *i* AX STAs, respectively, and note that these values are invariant for a certain *i*. Then, we express $T^{dl-ax}$ as follows:

(18) $$\begin{array}{cc} T^{dl-ax} & =\frac{1}{B}\sum_{i=1}^{B}T_{i}^{dl-ax}. \end{array}$$

For $T^{ul-ax}$, the probability $P_{i}^{\left(n,K\right)}$ is used:

(19) Tul−ax=∑n=0NaxNaxnbQbEax+bQnbEaxbEax+bQNax−n∑i=0BPi(n,K)Tiul−ax.

#### 4.3.2. Calculation of $E[L_{m}]$

Similarly to the idea above, we can express $E[L_{m}]$ as follows:

(20) $$\begin{array}{cc} & E\left[L_{ap}\right]=L\left(P^{dl-le}+P^{dl-ax}\cdot \frac{1}{B}\sum_{i=1}^{B}i\right)=L\left(P^{dl-le}+P^{dl-ax}\cdot \frac{1+B}{2}\right) \end{array}$$

(21) $$\begin{array}{cc} & E\left[L_{le}\right]=L\cdot P^{ul-le} \end{array}$$

(22) $$\begin{array}{cc} & E\left[L_{ax}\right]=L\cdot P^{ul-ax}\sum_{i=1}^{B}i\cdot P_{i}^{ul-ax}. \end{array}$$

For $E[L_{ap}]$, two transmission cases for LE STAs and AX STAs are considered. In particular, for AX STAs, the assumption in Section 4.1 is applied (i.e., $\frac{1}{B}\sum_{i=1}^{B}i$.), which means that the DL OFDMA frame size varies from *L* to B·L depending on the frame aggregation level. Similarly, for $E[L_{ax}]$, since 1 to *B* AX STAs could be involved in one OFDMA UL transmission, $\sum_{i=1}^{B}i\cdot P_{i}^{ul-ax}$ is applied.

### 4.4. Delay

As already adopted in many analytical studies [7,25,27,28], in this paper, the delay, which is defined as the time between the frame reaching the head of the transmission queue and the ACK frame being received, is used as a performance metric. Let $E[D_{y}]$ be the average delay of node *y*; it is then computed as follows:

(23) $$\begin{array}{c} E\left[D_{y}\right]=E\left[G_{y}\right]\cdot E\left[T_{y}^{delay}\right] \end{array}$$

where $E[G_{y}]$ is the average number of slots required to transmit a new frame and $E[T_{y}^{delay}]$ is the average length of those slots.

Unlike E[T] in Section 4.3, here only the slots that a tagged node has observed until successfully transmitting its frame should be taken into account. To do this, the conditional probability $P_{y}^{V}$ is defined to denote that node *y* observes slot *V* when its queue is not empty.

#### 4.4.1. Calculation of $E[T_{ap(le)}^{delay}]$

We first consider the case of the AP and the LE STAs. Similarly to the computation of E[T], $E[T_{y}^{delay}]$ is expressed as follows:

(24) $$\begin{array}{c} E[T_{ap}^{delay}]\\ =P_{ap}^{idle}T^{idle}+P_{ap}^{ul-le}T^{ul-le}+P_{ap}^{col}T^{col}+P_{ap}^{ul-ax}T^{ul-ax} \end{array}$$

(25) $$\begin{array}{c} E[T_{le}^{delay}]\\ =P_{le}^{idle}T^{idle}+P_{le}^{ul-le}T^{ul-le}+P_{le}^{dl-le}T^{dl-le}+P_{le}^{col}T^{col}+P_{le}^{ul-ax}T^{ul-ax}+P_{le}^{dl-ax}T^{dl-ax} \end{array}$$

For $P_{ap}^{idle}$ and $P_{ap}^{ul-le}$, the AP should not be in the (i,0) states (i.e., $\frac{1-b_{E_{ap}}-\tau_{ap}}{1-b_{E_{ap}}}$), while in the other cases, the AP should be in the (i,0) states. We then have the following:

(26) $$\begin{array}{cc} P_{ap}^{idle} & =\frac{1-b_{E_{ap}}-\tau_{ap}}{1-b_{E_{ap}}}{\left(1-\tau_{le}\right)}^{N_{le}} \end{array}$$

(27) $$\begin{array}{cc} P_{ap}^{ul-le} & =\frac{1-b_{E_{ap}}-\tau_{ap}}{1-b_{E_{ap}}}N_{le}\tau_{le}{\left(1-\tau_{le}\right)}^{N_{le}-1} \end{array}$$

(28) $$\begin{array}{cc} P_{ap}^{ul-ax} & =\frac{\tau_{ap}}{1-b_{E_{ap}}}{\left(1-\tau_{le}\right)}^{N_{le}}\cdot \frac{\lambda^{tf}}{\lambda_{ap}^{data}+\lambda^{tf}} \end{array}$$

(29) $$\begin{array}{cc} P_{ap}^{col} & =\frac{\tau_{ap}}{1-b_{E_{ap}}}\left(1-{\left(1-\tau_{le}\right)}^{N_{le}}\right)\\ & +\frac{1-b_{E_{ap}}-\tau_{ap}}{1-b_{E_{ap}}}\left(1-{\left(1-\tau_{le}\right)}^{N_{le}}-N_{le}\tau_{le}{\left(1-\tau_{le}\right)}^{N_{le}-1}\right). \end{array}$$

Similarly, we obtain the probabilities related to LE STAs as follows:

(30) $$\begin{array}{cc} P_{le}^{idle} & =\frac{1-b_{E_{le}}-\tau_{le}}{1-b_{E_{le}}}\left(1-\tau_{ap}\right){\left(1-\tau_{le}\right)}^{N_{le}-1} \end{array}$$

(31) $$\begin{array}{cc} P_{le}^{ul-le} & =\frac{1-b_{E_{le}}-\tau_{le}}{1-b_{E_{le}}}\left(1-\tau_{ap}\right)\left(N_{le}-1\right)\tau_{le}{\left(1-\tau_{le}\right)}^{N_{le}-2} \end{array}$$

(32) $$\begin{array}{cc} P_{le}^{ul-ax} & =\frac{1-b_{E_{le}}-\tau_{le}}{1-b_{E_{le}}}\cdot \tau_{ap}{\left(1-\tau_{le}\right)}^{N_{le}-1}\cdot \frac{\lambda^{tf}}{\lambda_{ap}^{data}+\lambda^{tf}} \end{array}$$

(33) $$\begin{array}{cc} P_{le}^{dl-le} & =\frac{1-b_{E_{le}}-\tau_{le}}{1-b_{E_{le}}}\cdot \tau_{ap}{\left(1-\tau_{le}\right)}^{N_{le}-1}\cdot \frac{\lambda_{ap}^{data}}{\lambda_{ap}^{data}+\lambda^{tf}}\cdot \frac{N_{le}}{N_{le}+N_{ax}} \end{array}$$

(34) $$\begin{array}{cc} P_{le}^{dl-ax} & =\frac{1-b_{E_{le}}-\tau_{le}}{1-b_{E_{le}}}\cdot \tau_{ap}{\left(1-\tau_{le}\right)}^{N_{le}-1}\cdot \frac{\lambda_{data}}{\lambda_{data}+\lambda_{tf}}\cdot \frac{N_{ax}}{N_{le}+N_{ax}} \end{array}$$

(35) $$\begin{array}{cc} P_{le}^{col} & =\frac{\tau_{le}}{1-b_{E_{le}}}\left(1-\left(1-\tau_{ap}\right){\left(1-\tau_{le}\right)}^{N_{le}-1}\right)\\ & +\frac{1-b_{E_{le}}-\tau_{le}}{1-b_{E_{le}}}\\ & \left(1-\left(1-\tau_{ap}\right){\left(1-\tau_{le}\right)}^{N_{le}-1}-\tau_{ap}{\left(1-\tau_{le}\right)}^{N_{le}-1}-\left(1-\tau_{ap}\right)\left(N_{le}-1\right)\tau_{le}{\left(1-\tau_{le}\right)}^{N_{le}-2}\right). \end{array}$$

For $E[G_{y}]$, let $G_{i}$ be the average number of slots required when a frame is successfully transmitted at backoff stage *i* (i.e., $E\left[G_{y}\right]=\sum G_{i}$.) Then, we have (subscripts omitted):

(36) $$G_{i}=\left(\frac{1+W_{i}}{2}\right)\left\{\begin{array}{cc} p^{i}, & i\in [0,m-1]\\ \frac{p^{i}}{1-p}, & i=m. \end{array}\right.$$

#### 4.4.2. Calculation of $E[T_{ax}^{delay}]$

We now consider the case of AX STAs. Based on the fact that until a successful frame transmission is achieved, the AX STA will repeat the transition between the *Q* state and the *U* state, $E[T_{ax}^{delay}]$ is expressed as follows:

(37) $$\begin{array}{cc} & E\left[T_{ax}^{delay}\right]=E\left[G_{ax,Q}\right]E\left[T_{ax,Q}^{delay}\right]+E\left[G_{ax,U}\right]E\left[T_{ax,U}^{delay}\right] \end{array}$$

where the subscripts *Q* and *U* are used to represent each case.

In the case of $E[T_{ax,Q}^{delay}]$, the tagged AX STA experiences all the slot cases except UL OFDMA. Thus, from Section 4.3, we have: $E\left[T_{ax,Q}^{delay}\right]=E\left[T^{-ul-ax}\right]$. To compute $E[G_{ax,Q}]$, we obtain the total number of slots required from $\frac{r+1}{r\left(1-c\right)}$. Since the portion of slots related to *Q* is $\frac{1}{r+1}$, we have $E\left[G_{ax,Q}\right]=\frac{1}{r\left(1-c\right)}$.

For $E[T_{ax,U}^{delay}]$, we need to consider the following two cases: (i) a tagged AX STA fails in the RU allocation phase (meaning that *c* is invoked); and (ii) a new frame arrives in the queue during the UL transmission of other AX STAs (i.e., *z*). For $E[G_{ax,U}]$, the number of slots required is $\frac{1}{1-c}$ for the first case and 1 for the second case (i.e., the initial state *U*), respectively. Putting this together, we have:

(38) $$\begin{array}{cc} E[T_{ax}^{delay}] & =\\ & \frac{b_{Q}}{b_{Q}+z\cdot b_{U}}\left(\frac{1}{r(1-c)}E\left[T_{ax,Q}^{delay}\right]+\frac{1}{1-c}E\left[T_{ax,U,c}^{delay}\right]\right)\\ & +\frac{z\cdot b_{U}}{b_{Q}+z\cdot b_{U}}\left(\frac{1}{r(1-c)}E\left[T_{ax,Q}^{delay}\right]+\frac{1}{1-c}E\left[T_{ax,U,c}^{delay}\right]+E\left[T_{ax,U,z}^{delay}\right]\right)\\ & =\frac{1}{r(1-c)}E\left[T_{ax,Q}^{delay}\right]+\frac{1}{1-c}E\left[T_{ax,U,c}^{delay}\right]+\frac{z\cdot b_{U}}{b_{Q}+z\cdot b_{U}}E\left[T_{ax,U,z}^{delay}\right]. \end{array}$$

where $E[T_{ax,U,c}^{delay}]$ and $E[T_{ax,U,z}^{delay}]$ is the average slot time for the case of *c* and *z*, respectively. Please note that these values can be obtained similar technique to computing Equations (7) and (19).

## 5. Performance Evaluation

### 5.1. Evaluation Setting

In this section, the analytical model is validated, and the performance of the networks is investigated via both numerical analysis and MATLAB simulation. The 802.11ax functionalities related to OFDMA MU transmission, such as RU contention, BSR, and TF, are implemented in the simulator on top of the legacy DCF. To configure the heterogeneous network environment, the parameter α is used to control the proportion of the AX STAs, i.e., $\alpha =\frac{N_{ax}}{N_{ax}+N_{le}}$. Each simulation is limited to 10 min and is run 100 times. The total throughput and the access delay of each node are used as performance metrics. The default simulation parameters are given in Table 1.

### 5.2. Model Validation

**Figure 6:**
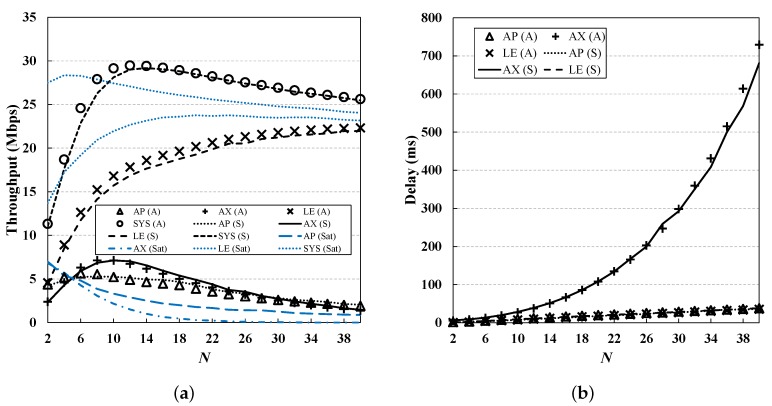
**Performance evaluation for different N values.** The throughput decreases as *N* increases, due to the heavy channel contention. For AX STAs, the access delay grows faster than for other STAs, as the TF transmission rate becomes low. (**a**) Throughput; (**b**) Delay.

**Figure 7:**
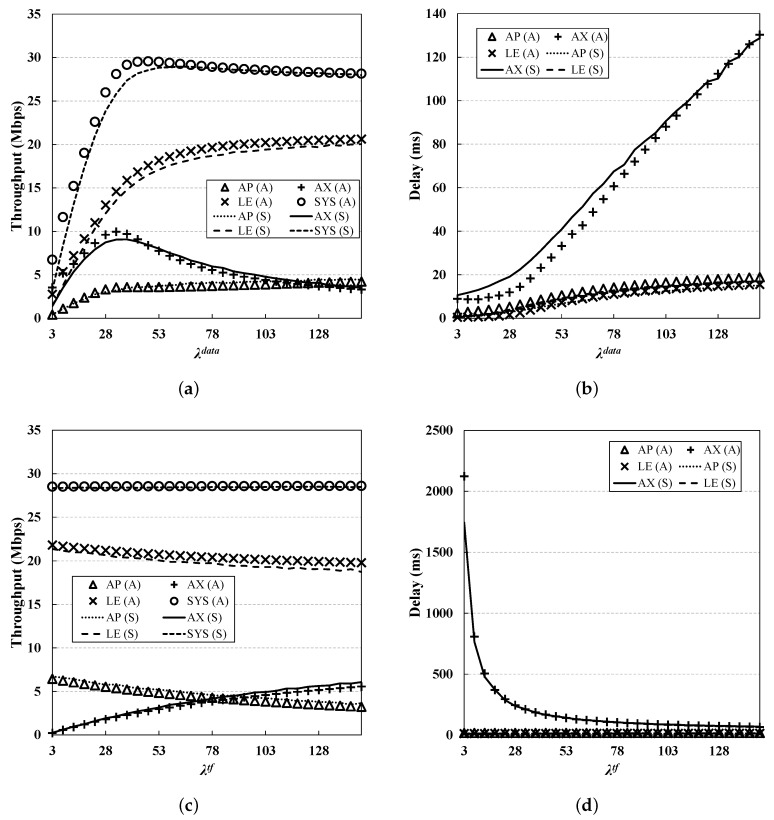
**Performance evaluation for different values of λ.** The performance of AX STAs is highly affected by $\lambda^{tf}$. (**a**) Throughput (varying $\lambda^{data}$); (**b**) Delay (varying $\lambda^{data}$); (**c**) Throughput (varying $\lambda^{tf}$); (**d**) Delay (varying $\lambda^{tf}$).

**Figure 8:**
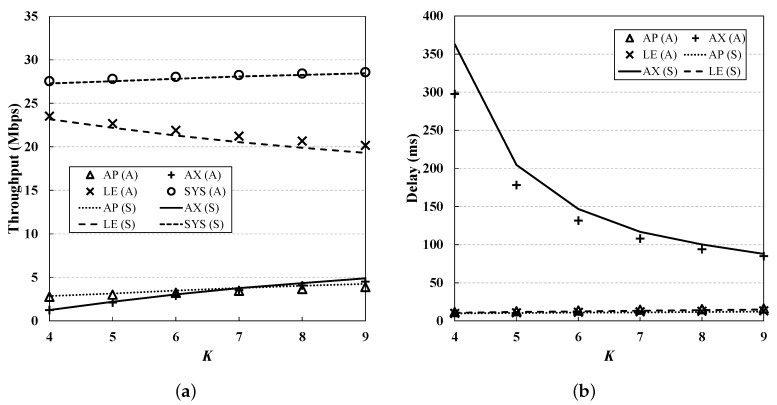
**Performance evaluation for different values of K.** Higher *K* values allow more AX STAs to transmit and receive concurrently, thus giving higher performance. (**a**) Throughput; (**b**) Delay.

**Figure 9:**
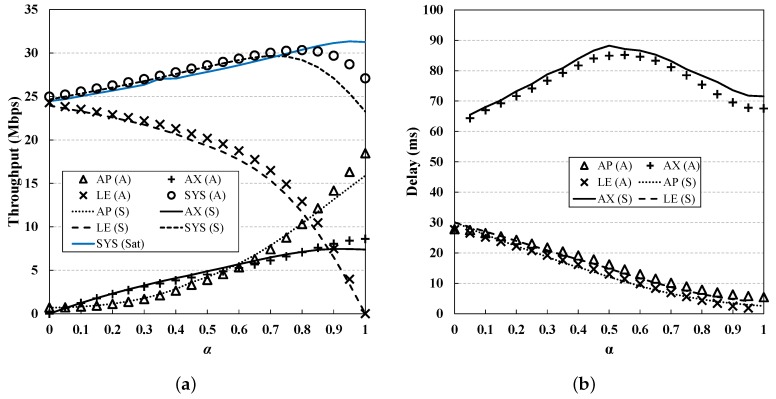
**Performance evaluation for different α values.** Both the throughput and delay performance improve as α increases, due to the higher efficiency of OFDMA MU transmissions. The system throughput in the case of $\lambda^{tf}=\lambda^{data}=250$ is depicted in the graph for comparison. (**a**) Throughput; (**b**) Delay.

**Figure 10:**
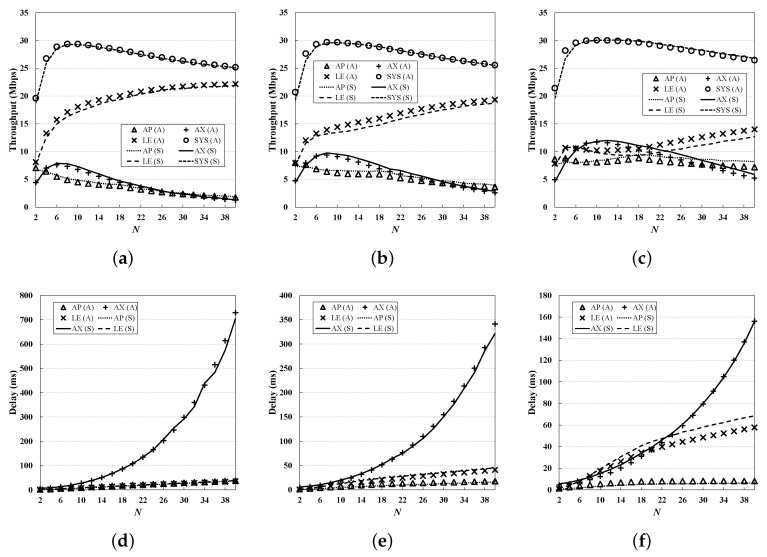
**Performance evaluation for different EDCA parameters.** Giving the high transmission priority to the AP results in that the AX STAs as well as the AP obtain a performance gain in term of both throughput and delay, at the expense of performance degradation of LE STAs. (**a**) Throughput ($W_{0}=32$, m=5); (**b**) Throughput ($W_{0}=16$, m=3); (**c**) Throughput ($W_{0}=8$, m=1); (**d**) Delay ($W_{0}=32$, m=5); (**e**) Delay ($W_{0}=16$, m=3); (**f**) Delay ($W_{0}=8$, m=1).

From Figure 6, Figure 7, Figure 8, Figure 9 and Figure 10, we observe that the results of simulation and numerical analysis match closely, confirming that our analytic model accurately captures the behavior of the 802.11ax MAC protocol under various network conditions. The purpose of each evaluation can be summarized as follows. Figure 6 shows the performance for changing numbers of STAs; Figure 7 illustrates the performance under non-saturated conditions by varying the frame arrival rates; Figure 8 shows the effect of RU contention in the network; and Figure 9 shows the influence of co-existence with non-802.11ax STAs in the network. The performance for changing EDCA parameter sets is depicted in Figure 10.

### 5.3. Varying *N*

Figure 6 shows the results for throughput and delay for different values of *N* from 2 to 40. For comparison, the throughput of the nodes in saturated condition (i.e., q=1) is added. Overall, as *N* increases, the throughput of the nodes decreases, due to the heavy channel contention. Although AX STAs do not contend for the channel in a random-access manner, the crowded condition of the LE STAs makes the probability of access to the AP lower; this results in lower throughput of the AP, and thus of the AX STAs, and also increases the access delay. In addition to the low transmission opportunity for TFs, the contention for OFDMA RUs of AX STAs becomes heavier as *N* increases; once an AX STA fails to obtain an OFDMA RU, it then needs to wait for another TF from the AP. In addition, since more sub-frames are associated within a single OFDMA transmission, the average OFDMA transmission time becomes longer, increasing the access delay.

For the LE STAs, the throughput continues to increase with *N* in the case of $\lambda^{data}=100$, while it becomes saturated as traffic load increases. We can see that the throughput for the LE STAs is higher than that for other nodes, and this has a great impact on the throughput of the system. This implies that the performance of AX STAs (especially when they co-exist with LE STAs) can be significantly reduced by the AP-dependent scheduling method of 802.11ax. It is, therefore, necessary to improve the MAC protocol to obtain the maximum OFDMA MU gain, and this issue is left to future work.

### 5.4. Varying λ

In this subsection, we investigate the performance for varying values of λ. The evaluation was conducted in two ways: varying only $\lambda^{data}$ and varying only $\lambda^{tf}$. Unless otherwise stated, the same value of $\lambda^{data}$ is applied to all nodes.

Figure 7a,b show the results when $\lambda^{data}$ is varied from 3 to 150 while $\lambda^{tf}$ is fixed at 100. In the case of the LE STA, the throughput first increases as $\lambda^{tf}$ increases, and then becomes saturated (at a throughput of 20 Mbps). Compared to the LE STAs, AX STAs are more sensitive to changes in $\lambda^{data}$. The throughput increases to about 9 Mbps, and then decreases again with $\lambda^{data}$. This is because as $\lambda^{data}$ increases, the transmission frequency of the TF decreases, and RU contention by AX STAs becomes heavier as the queue of AX STAs becomes saturated. This phenomenon is confirmed in Figure 7b, as the access delay increases significantly with $\lambda^{data}$.

Figure 7c,d show the results for settings that are opposite to those in the previous evaluation; here, $\lambda^{tf}$ is varied from 3 to 150, with $\lambda^{data}$ fixed at 100. Figure 7c shows that the LE STA is less affected by $\lambda^{tf}$ than in Figure 7a. As expected, as $\lambda^{tf}$ increases, the transmission opportunity of the AX STAs increases, and thus there is a performance gain in terms of delay and throughput. On the other hand, the performance of the AP is diminished due to the reduced transmission opportunities for its own data frames.

### 5.5. Varying *K*

The random-access-based RU contention may affect the performance of UL OFDMA for AX STAs. Figure 8 shows the evaluation results for different *K* values. As expected, we can see that the performance improves with increasing *K*; the throughput of the AX STAs increases and the delay decreases with *K*. Higher *K* values (i) allow more AX STAs to transmit and receive concurrently; and (ii) lower the BSR collision rate, increasing the MU UL transmission opportunity. This also helps to enhance the overall performance of other nodes, since the use of DL OFDMA transmission of the AP increases, and thus the AP consumes its transmission queue faster, reducing the channel contention.

### 5.6. Varying α

In this subsection, the network performance is investigated for various heterogeneous network configurations. Simulations are performed by changing α from 0 to 1, and Figure 9 shows the results. Overall, both the throughput and delay performance improve as α increases, but we can see that when α>0.8 the system throughput is rather diminished. This is because both the TF and data frame load are too low and thus the AX STAs do not obtain enough transmission opportunities. By contrast, in the case of $\lambda^{tf}=\lambda^{data}=250$, we can see the expected result: the larger the α value is, the higher the system throughput is.

When α is 1 (i.e., there are only AX STAs), both the AP and the AX STAs show the best performance. For the AP, frame collisions no longer arise in this scenario, and thus it achieves the highest throughput gain. On the other hand, when most nodes in the network are LE STAs, the throughput is bounded; this is as expected, since a higher proportion of the LE STAs means a lower channel access probability for the AP and thus also for the AX STAs. From Figure 9b, we can see that the delay initially increases for AX STAs. When α is small, even though the transmission opportunities are reduced for the AP, the RU contention of AX STAs is not so severe thus a small delay can be achieved; however, it begins to increase as α gradually increases. We can see that the delay decreases again at a value for α of 0.5; this is because the AP benefits from the low access delay for a small number of LE STAs, and can deliver TFs to the network more frequently.

### 5.7. Varying EDCA Parameter Sets

As shown in the previous set of simulations, AX STAs may suffer from the unfairness problem, since it can access the medium only after the AP gets the transmission opportunity. As mentioned earlier, one effective way to address this problem is to adopt different transmission priorities to nodes, and EDCA can be used here [10,18,19,25,27]. In this subsection, the network performance is investigated for different EDCA parameter sets; simulations are performed by changing minimum contention window (i.e., $W_{0}$) and maximum contention stage (i.e., *m*) of the AP, as (32, 5), (16, 3), and (8, 1). Figure 10 shows the result. As expected, as the channel access priority of the AP increases, both the AP and the AX STAs obtain more performance gains in terms of throughput and delay. In particular, from Figure 10c we can see that the system throughput is highest when the AP’s channel access priority is highest, at the expense of significant performance degradation of LE STAs. This result shows that it is possible to balance the transmission of nodes by simply adjusting the existing EDCA parameters; however, to further enhance the performance gain, careful design of algorithms and protocols that exploit several advanced techniques such as user scheduling and MIMO is required. The study on this is left for future work.

## 6. Conclusions

This paper provides a numerical analysis of the 802.11ax MAC protocol under non-saturated traffic conditions. Co-existence with non-802.11ax devices is taken into account in the analysis, to investigate the network performance for various heterogeneous network circumstances. Both analytical and simulation evaluations verify the proposed model under non-saturated conditions, and the MAC protocol is evaluated for various network parameters.

## Figures and Tables

**Table 1:** Default Simulation Parameters.

Parameter	Value
$W_{0}$	32
*m*	5
*L*	6000 Bytes
Bandwidth	20 MHz
Data Rate	36 Mbps
Basic Rate	6.5 Mbps
*N*	20
α	0.5
$\lambda^{data}$	100 frames/s
$\lambda^{tf}$	100 frames/s
*K*	9
$T^{idle}$	20 μs
$T^{col}$	1458 μs
$T^{ul-le}$	1478 μs
$T^{dl-le}$	1478 μs
$T_{1}^{dl-ax}$	1478 μs
$T_{0}^{ul-ax}$	341.574 μs
$T_{1}^{ul-ax}$	1675 μs
